# Supplementary material for: Meta-Analysis and Gene Set Enrichment Relative to ER Status Reveal Elevated Activity of MYC and E2F in the “Basal” Breast Cancer Subgroup
Source: PLoS One. 2009 Mar 9;4(3):e4710. doi: 10.1371/journal.pone.0004710 (PMC2650420; doi:10.1371/journal.pone.0004710)
Supplement: Dataset S1 — GSEA results from first screen. The file contains a zipped archive of the HTML results of the second GSEA screen. Reports for enriched or depleted datasets may be accessed from the “index.html” link within the my_analysis.GseaPreranked.1219975950625.rpt.zip ZIP archive. Enrichment plots are available for each of the 50 most enriched and depleted gene sets. (13.32 MB ZIP) [file pone.0004710.s001.zip › my_analysis.GseaPreranked.1219975950625.rpt.zip!//VANTVEER_BREAST_OUTCOME_GOOD_VS_POOR_DN.html]

Details for gene set VANTVEER\_BREAST\_OUTCOME\_GOOD\_VS\_POOR\_DN[GSEA]

|  || Dataset | zSco.ERneg.to.ERpos\_collapsed |
| Phenotype | NoPhenotypeAvailable |
| Upregulated in class | na\_pos |
| GeneSet | VANTVEER\_BREAST\_OUTCOME\_GOOD\_VS\_POOR\_DN |
| Enrichment Score (ES) | 0.7260185 |
| Normalized Enrichment Score (NES) | 2.7483256 |
| Nominal p-value | 0.0 |
| FDR q-value | 0.0 |
| FWER p-Value | 0.0 |
Table: GSEA Results Summary

  

Fig 1: Enrichment plot: VANTVEER\_BREAST\_OUTCOME\_GOOD\_VS\_POOR\_DN      
 Profile of the Running ES Score & Positions of GeneSet Members on the Rank Ordered List

  

| PROBE | GENE SYMBOL | GENE\_TITLE | RANK IN GENE LIST | RANK METRIC SCORE | RUNNING ES | CORE ENRICHMENT || 1 | CTSL2 |  |  | 1 | 7.773 | 0.0351 | Yes |
| 2 | ORC6L |  |  | 33 | 6.507 | 0.0622 | Yes |
| 3 | BUB1 |  |  | 41 | 6.445 | 0.0909 | Yes |
| 4 | CENPA |  |  | 51 | 6.315 | 0.1188 | Yes |
| 5 | TMEFF1 |  |  | 67 | 6.085 | 0.1452 | Yes |
| 6 | NMB |  |  | 71 | 6.063 | 0.1724 | Yes |
| 7 | TRIP13 |  |  | 96 | 5.890 | 0.1973 | Yes |
| 8 | CTPS |  |  | 123 | 5.706 | 0.2212 | Yes |
| 9 | GGH |  |  | 161 | 5.504 | 0.2433 | Yes |
| 10 | MCM6 |  |  | 198 | 5.317 | 0.2646 | Yes |
| 11 | BIRC5 |  |  | 229 | 5.191 | 0.2859 | Yes |
| 12 | CKS2 |  |  | 239 | 5.145 | 0.3085 | Yes |
| 13 | TFRC |  |  | 259 | 5.071 | 0.3300 | Yes |
| 14 | CENPF |  |  | 268 | 5.028 | 0.3522 | Yes |
| 15 | EZH2 |  |  | 312 | 4.888 | 0.3710 | Yes |
| 16 | MCCC1 |  |  | 326 | 4.861 | 0.3921 | Yes |
| 17 | CDC25B |  |  | 340 | 4.826 | 0.4129 | Yes |
| 18 | RFC4 |  |  | 370 | 4.741 | 0.4322 | Yes |
| 19 | CCNB2 |  |  | 431 | 4.589 | 0.4485 | Yes |
| 20 | MAD2L1 |  |  | 477 | 4.446 | 0.4652 | Yes |
| 21 | MAPRE2 |  |  | 507 | 4.333 | 0.4826 | Yes |
| 22 | RAB6B |  |  | 517 | 4.314 | 0.5015 | Yes |
| 23 | NMU |  |  | 520 | 4.311 | 0.5208 | Yes |
| 24 | PRC1 |  |  | 543 | 4.253 | 0.5384 | Yes |
| 25 | ASNS |  |  | 618 | 4.099 | 0.5514 | Yes |
| 26 | EXT1 |  |  | 673 | 3.986 | 0.5654 | Yes |
| 27 | PCTK1 |  |  | 708 | 3.915 | 0.5805 | Yes |
| 28 | HRB |  |  | 752 | 3.840 | 0.5947 | Yes |
| 29 | PIR |  |  | 757 | 3.834 | 0.6117 | Yes |
| 30 | CA9 |  |  | 909 | 3.623 | 0.6167 | Yes |
| 31 | SLC2A3 |  |  | 934 | 3.575 | 0.6311 | Yes |
| 32 | MTMR2 |  |  | 965 | 3.531 | 0.6448 | Yes |
| 33 | SACS |  |  | 1039 | 3.426 | 0.6548 | Yes |
| 34 | ADM |  |  | 1046 | 3.419 | 0.6699 | Yes |
| 35 | COL4A2 |  |  | 1108 | 3.351 | 0.6804 | Yes |
| 36 | PFKP |  |  | 1165 | 3.281 | 0.6911 | Yes |
| 37 | FBXO5 |  |  | 1304 | 3.137 | 0.6949 | Yes |
| 38 | CP |  |  | 1460 | 2.987 | 0.6967 | Yes |
| 39 | FLT1 |  |  | 1486 | 2.957 | 0.7082 | Yes |
| 40 | GMPS |  |  | 1543 | 2.907 | 0.7171 | Yes |
| 41 | PTDSS1 |  |  | 1806 | 2.686 | 0.7095 | Yes |
| 42 | MMP9 |  |  | 1891 | 2.616 | 0.7150 | Yes |
| 43 | ECT2 |  |  | 2026 | 2.509 | 0.7163 | Yes |
| 44 | RRM2 |  |  | 2047 | 2.485 | 0.7260 | Yes |
| 45 | ESM1 |  |  | 2326 | 2.308 | 0.7155 | No |
| 46 | NDRG1 |  |  | 2463 | 2.218 | 0.7153 | No |
| 47 | BNIP3 |  |  | 2662 | 2.111 | 0.7099 | No |
| 48 | TK1 |  |  | 2730 | 2.076 | 0.7142 | No |
| 49 | PSMD2 |  |  | 2759 | 2.063 | 0.7215 | No |
| 50 | CCNE2 |  |  | 3424 | 1.738 | 0.6793 | No |
| 51 | RAD21 |  |  | 3642 | 1.642 | 0.6703 | No |
| 52 | VEGF |  |  | 3911 | 1.549 | 0.6571 | No |
| 53 | PRAME |  |  | 3945 | 1.537 | 0.6616 | No |
| 54 | GNAZ |  |  | 4789 | 1.226 | 0.6036 | No |
| 55 | GBE1 |  |  | 5011 | 1.151 | 0.5921 | No |
| 56 | ST7 |  |  | 5347 | 1.027 | 0.5715 | No |
| 57 | PGK1 |  |  | 5836 | 0.865 | 0.5386 | No |
| 58 | PSMD7 |  |  | 6263 | 0.727 | 0.5098 | No |
| 59 | STK3 |  |  | 7585 | 0.275 | 0.4114 | No |
| 60 | DCK |  |  | 8935 | -0.280 | 0.3109 | No |
| 61 | STX1A |  |  | 9880 | -0.771 | 0.2432 | No |
| 62 | MGAT4A |  |  | 10559 | -1.215 | 0.1975 | No |
| 63 | IGFBP5 |  |  | 11829 | -2.352 | 0.1125 | No |
Table: GSEA details [plain text format]

  

Fig 2: VANTVEER\_BREAST\_OUTCOME\_GOOD\_VS\_POOR\_DN: Random ES distribution      
 Gene set null distribution of ES for **VANTVEER\_BREAST\_OUTCOME\_GOOD\_VS\_POOR\_DN**
